# Draft genome sequencing data of a pathogenic *Pantoea stewartii* subspecies *stewartii* strain SQT1 causing bronzing disease of jackfruit in Malaysia

**DOI:** 10.1016/j.dib.2020.105634

**Published:** 2020-04-30

**Authors:** Rohaya Ibrahim, Noor Wahida Ismail-Suhaimy, Tan Shu-Qing, Siti Izera Ismail, Md Yasin Ina-Salwany, Mohd Termizi Yusof, Mansor Hakiman, Daljit Singh Karam, Dzarifah Zulperi

**Affiliations:** a Department of Plant Protection, Faculty of Agriculture, Universiti Putra Malaysia, 43400 Serdang, Selangor, Malaysia; b Department of Aquaculture, Faculty of Agriculture, Universiti Putra Malaysia, 43400 Serdang, Selangor, Malaysia; c Department of Crop Science, Faculty of Agriculture, Universiti Putra Malaysia, 43400 Serdang, Selangor, Malaysia; d Department of Land Management, Faculty of Agriculture, Universiti Putra Malaysia, 43400 Serdang, Selangor, Malaysia; e Department of Microbiology, Faculty of Biotechnology and Biomolecular Sciences, Universiti Putra Malaysia, 43400 Serdang, Selangor, Malaysia; f Laboratory of Sustainable Resources Management, Institute of Tropical Forestry and Forest Products, Universiti Putra Malaysia, 43400, Serdang, Selangor, Malaysia

**Keywords:** Malaysia, bronzing disease, jackfruit, *Pantoea stewartii* subspecies *stewartii*, genome sequencing, Illumina Hiseq, virulence factors, T6SS

## Abstract

A Gram-negative bacterium, *Pantoea stewartii* subspecies *stewartii* (*P. stewartii* subsp. *stewartii*) has been recognized as the causative agent for jackfruit bronzing disease in Malaysia. Here, we report the whole genome sequencing dataset of *P. stewartii* subsp. *stewartii* strain SQT1 isolated from local infected jackfruit. The paired-end libraries with an insert size of 350 bp was subjected to the Illumina Hiseq 4000, generating a genome size of 4,783,993 bp with a G+C content of 53.7%. A total protein of 4,671 was identified including virulence factors, resistance factors and secretion systems. *Pantoea stewartii* subsp. *stewartii* strain DC283 (NCBI accession no. CP017581.1) was used as a reference genome, where the query hit 72% coverage and average sequencing depth of 68. In total, 28,717 nucleotide polymorphisms, 520 small insertion/deletions and 142 structure variants were identified. The complete genome was deposited at the European Nucleotide Archive under the sample accession number ERP119356 and study accession number PRJEB36196.

**Specifications Table**

**Table:** 

**Subject**	Molecular Biology
**Specific subject area**	Microbial genomics
**Type of data**	Table Figure Completed genome sequence in FASTA format
**How data were acquired**	Illumina Hiseq sequencing platform
**Data format**	Raw and analyzed
**Parameters for data collection**	Genomic DNA from bacterial pure culture
**Description of data collection**	Bacterial isolation from pure culture, sequencing the whole genome, genome assembly by reference method and annotation
**Data source location**	City/Town/Region: Muadzam Shah State: Pahang Country: Malaysia
**Data accessibility**	Repository name: European Nucleotide Archive Data identification number: ERP119356 Direct URL to data: 1) https://www.ebi.ac.uk/ena/browser/view/PRJEB36196 2) https://www.ebi.ac.uk/ena/browser/text-search?query=PRJEB36196
**Related research article**	Zulperi, D., Manaf, N., Ismail, S. I., Karam, D. S., & Yusof, M. T. (2017). First report of *Pantoea stewartii* subspecies *stewartii* causing fruit bronzing of jackfruit (*Artocarpus heterophyllus*), a new emerging disease in Peninsular Malaysia. Plant Disease, 101(5), 831-831. https://doi.org/10.1094/PDIS-11-16-1689-PDN. 1 Ibrahim, R., Ismail-Suhaimy, N. W., Shu-Qing, T., Ismail, S. I., Abidin, N., Hakiman, M., Karam, D. S., Ahmad-Hamdani, M. S., Yusof, M. T. and Zulperi, D. (2019). Molecular characterization and phylogenetic analysis of *Pantoea stewartii* subspecies *stewartii* causing bronzing disease of jackfruit in Malaysia based on cps and hrp gene sequences. Journal of Plant Pathology, 1-7. https://doi.org/10.1007/s42161-019-00383-7.

**Value of the Data**

The draft genome sequence data of *P. stewartii* subsp. *stewartii* strain SQT1 provides additional information to the available limited number of *P. stewartii* genomes in the public databases and helps for better understanding on bacterial interaction with the host genes resulting in enhanced breeding programs with resistance to jackfruit bronzing disease.
Virulence and antibiotic resistance gene analysis may predict an organism's likelihood of being a multidrug resistance pathogen.
Data can be used as reference to other *P. stewartii* genomes to provide insights into the possible mechanisms of virulence.

## 1 Data Description

Bronzing disease is a serious problem in Malaysia's jackfruit industry, as it decreases the price of fresh jackfruit and consumer preferences. The bronzing disease causal bacterium, *P. stewartii* subsp. *stewartii* strain SQT1 was isolated from infected jackfruit in Muadzam Shah, Pahang, Malaysia [1,2]. In this data, genomic sequences were generated and assembled before proceeded to annotation process. Annotation by Rapid Annotation using Subsystem Technology (RAST) revealed that the final assembly of the draft genome consists of 4,783,993 bp genome length with 53.7% G+C content, 307,002 N50, and 67 contigs (Table 1).

**Table 1:** Overview of *Pantoea stewartii* subspecies *stewartii*, strain SQT1.

Attribute	Value
Genome size (bp)	4,783,993
G + C content (%)	53.7
N50	307,002
Contigs	67
Subsystems	517
Coding sequences	4609
RNAs	71

**Fig. 1:**
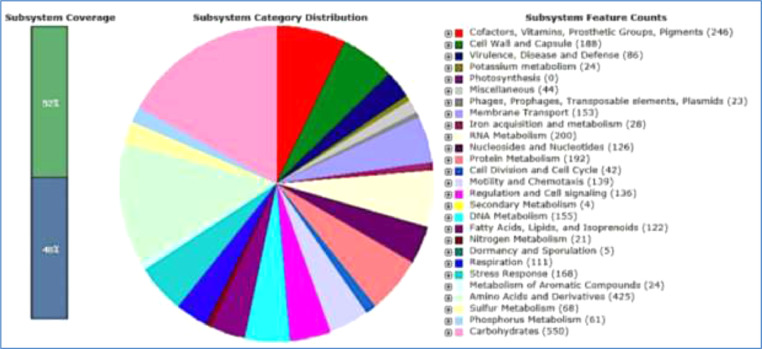
Subsystem coverage, category distribution and features counts of the *Pantoea stewartii* subspecies stewartii strain SQT1.

RAST also disclosed the subsystem coverage, category distribution and features counts of the draft genome as depicted in Figure 1. The subsystem coverage comprised 52% of the protein, with 517 subsystem, 4609 protein coding sequences and 71 RNAs (Table 1). The subsystem feature counts pointed out that 86 of the coding sequences related to virulence, disease and defense. Under the adhesion subdivision, a conserved genomic region-encoded YidE gene was presented and acting as a mediator on hyper-adherence.

The genome organism is found to be tolerant towards colicin E2, a type of bacteriocin that causes DNA breakdown as their modes of action [3]. It also encodes for antimicrobial resistance gene against fluoroquinolone. The genome has a beta-lactamase which is a defining feature of the organism. RAST server also uncovered the resistance towards heavy metals; cobalt-zinc-cadmium, arsenic and copper. The mycobacterium virulence operon was also identified to be responsible for the invasion and intracellular resistance of the bacterial genome.

There were 153 coding sequences (CDS) related to protein secretion systems (Figure 1), and type VI secretion system (T6SS) was identified as the main bacterial secretion machinery. Of 34 T6SS, four VgrG proteins were found on the genome sequences at locations 2657, 2667, 2671 and 3078. Together with Hcp, VgrG protein forms a tube of stacked hexamers in vitro, a hallmark of T6SS [4]. T6SS has been reported in plant pathogen of Pantoea species [5], [6], [7] and within α, β and γ-proteobacteria are mostly reported [8]. Previous studies have shown that T6SS engages in manipulation of host cell and interbacterial competition by injecting toxins effectors into prokaryotic or eukaryotic cells. The role is not restricted to virulence and antibacterial activity since the system is dispersing among the commensal and pathogenic phytobacteria, thus adding the colonization benefits and fitness *in planta* [9]. T6SS could be a dynamic system for bacterial communication by cell-to cell signalling system [10], since the T6SS components that have been used for destroying cells can be reused for new T6SS assembly [11].

## 2 Experimental design, materials, and methods

### 2.1 DNA extraction

Following sample preparation, total genomic DNA of strain SQT1 was extracted using the Presto™ mini gDNA Bacteria Kit (Geneaid Biotech Ltd., Taiwan) following manufacturer's instructions. For the sample quality control, DNA quality was measured using a NanoDrop spectrophotometer (Thermo Scientific, USA) and proceeded to library preparation and sequencing analyses.

### 2.2 Library preparation and sequencing

The method of DNA library was performed according to Wang et al. [12]. Fragmentation of the genomic DNA by random nebulisation was achieved using a paired‑end DNA sample preparation kit. The process resulted in double‑stranded DNA fragments consisting of 3′ or 5′ overhangs. The overhangs were transformed to blunt ends, where the 3′ to 5′ exonuclease activity removed 3′ overhangs and the polymerase activity filled in the 5′ overhangs. Using the polymerase activity, an ‘A’ base has been linked to the 3’ end. Ligation of the DNA adaptors and the DNA fragments took place prior to purification to remove unligated adapters, as well as self-ligated adapters. The products were subjected to quality control and detected the fragments size and yield. The generated library was analysed using Hiseq 4000 platform (Illumina, Inc.) that utilises sequencing‑by‑synthesis technology.

### 2.3 Bioinformatics analysis

The sequencing data (raw data) generated from the Illumina pipeline was analysed using CASAVA base calling. Data quality control was carried out where the adapter sequence and low quality reads were cut off and generated clean data, where Burrows‑Wheeler Aligner (BWA) [13] was used to align reads to the reference sequence, *P. stewartii* subs. *stewartii* strain DC283, whilst coverage was computed by SAMTOOLS software. The aligned data was stored in BAM files and ready for variant calling analyses.

### 2.4 Data quality control

Quality control was performed and the adapter and low quality sequences were removed. Also, the reads with unknown bases of greater than 10% were removed. Statistical analysis was conducted afterwards to get the raw FASTQ data as well as clean data. The obtained clean data was used for subsequent analysis.

### 2.5 SNPs/InDel analysis

Not only detecting individual single nucleotide polymorphisms (SNPs), SAMtools software was also used to detect insertion and deletion (InDel) of small fragments that are less than 50bp. Then, the position of SNP/InDel in the functional regions of the SQT1 genome was annotated.

### 2.6 SV analysis

The insertion, deletion, inversion, intra-chromosomal translocation, and inter-chromosomal translocation among the reference and sample were performed by BreakDancer software.

## Declaration of Competing Interest

The authors declare that they have no known competing financial interests or personal relationships that could have appeared to influence the work reported in this paper.

## Appendix Supplementary materials

Image, application 1
